# Anti-Inflammatory Klotho Protein Serum Concentration Correlates with Interferon Gamma Expression Related to the Cellular Activity of Both NKT-like and T Cells in the Process of Human Aging

**DOI:** 10.3390/ijms24098393

**Published:** 2023-05-07

**Authors:** Lucyna Kaszubowska, Jerzy Foerster, Jan Jacek Kaczor, Mateusz Jakub Karnia, Zbigniew Kmieć

**Affiliations:** 1 Department of Histology, Medical University of Gdańsk, Dębinki 1, 80-211 Gdańsk, Poland; 2 Department of Social and Clinical Gerontology, Medical University of Gdańsk, Dębinki 1, 80-211 Gdańsk, Poland; 3 Department of Animal and Human Physiology, University of Gdańsk, J. Bażyńskiego 8 Street, 80-308 Gdańsk, Poland

**Keywords:** CD3+CD56+ cells, NKT-like cells, CD3+ cells, T cells, immune homeostasis, interferon gamma, Klotho, aging, interleukin 6, tumor necrosis factor

## Abstract

Klotho is a beta-glucuronidase that reveals both anti-inflammatory and anti-oxidative properties that have been associated with mechanisms of aging. The study aimed to analyze the relationships between the serum concentration of soluble α-Klotho and cellular activity of two populations of lymphocytes; T and NKT-like cells corresponding to the level of cytokine secretion; i.e., IFN-γ, TNF-α, and IL-6. The studied population comprised three age groups: young individuals (‘young’), seniors aged under 85 (‘old’), and seniors aged over 85 (‘oldest’). Both NKT-like and T cells were either non-cultured or cultured for 48 h and stimulated appropriately with IL-2, LPS or PMA with ionomycin to compare with unstimulated control cells. In all studied age groups non-cultured or cultured NKT-like cells revealed higher expressions of TNF-α, IL-6, and IFN-γ than T cells. α-Klotho concentration in serum decreased significantly in the process of aging. Intriguingly, only IFN-γ expression revealed a positive correlation with α-Klotho protein serum concentration in both non-cultured and cultured T and NKT-like cells. Since IFN-γ is engaged in the maintenance of immune homeostasis, the observed relationships may indicate the involvement of α-Klotho and cellular IFN-γ expression in the network of adaptive mechanisms developed during the process of human aging.

## 1. Introduction

Aging is a progressive and multifactorial physiological process characterized by an accumulation of impairments in various cellular and molecular structures, leading to a gradual decline in the adaptability and resistance to disparate cellular stresses [1,2]. It is accompanied by the increase in oxidative stress due to the dysfunction of mitochondria producing raised amounts of reactive oxygen species (ROS) and impaired with age cellular mechanisms of anti-oxidative protection which result in the shift of the redox balance towards a pro-oxidative state [3]. Aging is also characterized by the disturbed equilibrium between pro-inflammatory and anti-inflammatory processes which results in a chronic low-grade state of inflammation described as inflamm-aging [4]. Moreover, the inflammatory response can be activated by oxidative stress and these two processes make up a specific type of synergistic association that contributes to the low-grade inflammation state characteristic for the process of aging [5]. The immune response is then chronically activated and increased serum levels of pro-inflammatory cytokines; i.e., TNF-α, IL-1, and IL-6 cause the progressive activation of leukocytes and modify numerous cellular functions [6]. The immune homeostasis is, therefore, dysregulated which results in aging-associated failure of immune response termination [7].

Inflamm-aging also results from a disrupted equilibrium between the function of innate and adaptive immune systems [8]. Innate immunity starts to reveal a mild hyperactivity of circulating inflammatory factors, whereas adaptive immunity declines resulting in the progression of adaptive immunosenescence [9]. The senescent cells stimulate the secretion of pro-inflammatory cytokines [10,11]. The initialized anti-inflammatory mechanisms are usually insufficient to curb the improperly active innate immunity which results in the inflamm-aging process. However, recent data indicate that age-related immune changes seem to present rather a mix of functional and dysfunctional alterations which may be both adaptive and maladaptive in the process of aging [12].

Klotho (α-Klotho) is a beta-glucuronidase associated with the mechanisms of aging [13]. The full-length membrane form of the protein is composed of a large extracellular domain, a membrane-spanning segment, and a short intracellular domain [14]. The full-length soluble Klotho comprised two homologous domains KL1 and KL2 is generated by metalloproteinases, ADAM-10 and ADAM-17 or β-secretase 1 (BACE1), as a result of α-cut at the transmembrane portion of the protein [15]. Klotho was described initially to be produced mainly in the kidneys; however, its expression was also found in a range of human tissues and organs, including arteries, epithelia, endocrine glands, placenta, lung, adipose tissue, certain brain regions, muscle tissue, digestive tract, lymph nodes, bone marrow [16,17], and blood cells, including lymphocytes and monocytes [18,19]. The soluble α-Klotho protein is the most abundant form found in blood, urine, and cerebrospinal fluid which reveals hormone-like activity affecting the process of ageing in tissues in which this protein is not expressed [14,20]. It regulates multiple signaling pathways involving MAPK/ERK, ASK1/p38 MAPK, cAMP/PKA, Nrf1/2, insulin/IGF-1/PI3K, caspase 3/caspase 9, and p53/p21 [14,21].

The anti-inflammatory effect of α-Klotho protein includes decrease in the expression of pro-inflammatory cytokines [22], increased secretion of anti-inflammatory factors [19], and reduction of oxidative stress molecules involving the PI3K/AKT pathway and Nrf2/HO-1 pathway (heme oxygenase pathway) [23,24]. This group of anti-inflammatory activities concern also the inhibition of NF-κB (nuclear factor kappa-light-chain-enhancer of activated B cells) or NLRP3 inflammasome signaling pathways [25,26]. Moreover, pro-inflammatory processes were found to negatively modulate Klotho expression, implying the existence of feedback control system(s) [27].

Aging of the immune system has been regarded as both remodeling of the immune repertoire and the process unraveling mechanisms of immune plasticity crucial for the maintenance of immune homeostasis [28]. It is characterized by the shrinkage of the lymphocyte repertoire, including B and T cells, and subsequent progression of immunosenescence [29,30]. Aging B cells display deficiencies in BCR (B cell receptor) signaling and processes of proliferation and maturation [30,31] as well as some inefficiencies in immunoglobulin recombination and isotype switching [32]. Aging T cells present ineffective triggering of the TCR-CD3 complex deteriorated by down-regulation of CD4 and CD8 coreceptors [9]. In consequence, T cells show numerous defects in cytokine production and loss of classical T-helper and T-cytotoxic functions [30,33]. The process of T cell senescence is also characterized by the progressive loss of CD28 expression and de novo acquisition of a diverse array of receptors normally expressed on NK cells [28,34]. Cells phenotypically characterized by the co-expression of TCR (T cell receptor) with NK-related receptors (NKRs) were described as NK-like T cells [35], innate-like T cells, or NKT-like cells [36,37], contrary to classical NKT (‘invariant NKT’; iNKT) cells revealing expression of invariant TCRs (Vα24Jα18) that bind glycolipids presented by the CD1d molecule [38]. Invariant NKT cells account for only 0.1–1% of T lymphocytes in human peripheral blood [39], whereas NKT-like cells comprise approximately 5–15% of the peripheral T cell population [36]. Moreover, iNKT and NKT-like lymphocyte subsets are differentially affected by the process of human aging; i.e., the percentage of peripheral blood iNKT cells decreases with age in contrast to the increased frequency of peripheral blood NKT-like cells [38]. This may suggest that CD3+CD56+ cells play an important role in the control of the process of aging. They can be involved also in prevention of diseases by elimination of infected cells or tumor cells due to their cytotoxic and cytokine-secreting abilities. On the other hand, their accumulation may be detrimental to immune system homeostasis and may result in the development of autoimmune diseases and chronic inflammation [37].

It has been shown that binding to some of the stimulatory receptors, i.e., CD56 or NKG2D, independent of TCR ligation, resulted in NK-like T cell activation and various cellular outcomes, including expression of activation antigens (CD25, CD69) and synthesis of cytolytic proteins [28]. However, a domination of inhibitory NKRs on aged T cells could either designate a continued malfunction of the aged T cells or possibly an adaptation to control autoreactive cells in old age [28,40]. Such adaptation of the aging immune system can help to maintain immune homeostasis despite the inefficiency of classical TCR signaling and the contraction of diversity of TCR repertoire [28]. Moreover, the acquisition of innate characteristics by T cells may present the process of remodeling of immune system, leading to successful aging [37].

Tumor necrosis factor (TNF-α) is a pleiotropic cytokine involved in the control of inflammation, anti-tumor responses, and maintenance of immune system homeostasis [41]. It is a product of the effector CD4 and CD8 T cells or innate cells that can moderate the T cell response and function of the other immune cell types [42]. The CD3+CD56+ cells share NK and T cell characteristics; i.e., they produce TNF-α and IFN-γ after activation [43].

Interleukin 6 (IL-6) is referred to as a pleiotropic cytokine that in the immune system acts on numerous cell types and influences multiple processes. It can be produced and secreted by many cell types, including fibroblasts, endothelial cells, T cells, and innate immune cells, including circulating dendritic cells, neutrophils, NK cells, monocytes, eosinophils, basophils, tissue-resident mast cells, and macrophages [44,45]. IL-6 was found to activate a variety of inflammatory processes in hematopoietic cells and downregulate IFN-γ expression or cytotoxic responses in CD8 T cells and NK cells [46,47].

Interferon gamma is a pleiotropic, immunomodulatory cytokine that effects both innate and adaptive immunity. It is secreted by activated CD4, CD8, and gamma delta T lymphocytes as well as NK and NKT cells. Additional sources include B lymphocytes and antigen-presenting cells [48]. This cytokine in the inflammatory environment triggers the activation of the immune response and stimulates the elimination of pathogens. It also prevents overactivation of the immune system and subsequent tissue damage [49]. Interferon gamma was also shown to be involved in the maintenance of tissue homeostasis to balance immune response and immune tolerance in healthy tissues [50].

The study aimed to find associations between serum levels of soluble α-Klotho, an anti-inflammatory and anti-oxidative protective protein, and cellular activity of two populations of lymphocytes; T (CD3+) and NKT-like (CD3+CD56+) cells corresponding to the level of cytokine expression; i.e., IFN-γ, a cytokine, revealing both pro- and anti-inflammatory properties [51] and inflammatory cytokines, TNF-α and IL-6 [52].

## 2. Results

### 2.1. Content of Sulfhydryl (-SH) Groups in Serum Samples

**Figure 1:**
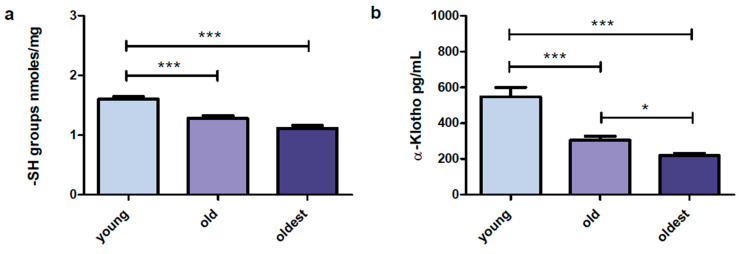
Selected parameters determined in sera of the young, the old aged under 85 and the oldest (aged over 85). Data are presented as mean ± SEM. Solid horizontal lines denote statistically significant differences found between the studied age groups. The respective symbols denote * *p* < 0.05, *** *p* < 0.001. (**a**) Serum content of sulfhydryl (-SH) groups presented in nmoles/mg of protein in the serum sample. (**b**) α-Klotho serum concentration (pg/mL).

The serum concentration of protein sulfhydryl groups was measured in all studied age groups (young, old, and the oldest). The highest concentration was found in the young and it was significantly higher compared to the old and the oldest. Simultaneously, both senior groups did not differ in the –SH group content (Figure 1a).

### 2.2. α-Klotho Concentration in Serum Samples

The concentration of α-Klotho protein was analyzed in the sera of the oldest, old, and young subjects. The highest concentration of this protein was found in the young and it was nearly twice as high than observed in the old, and it was 2.5 times higher compared to the oldest. Moreover, α-Klotho serum concentration in the group of the old was significantly higher compared to the group of the oldest (Figure 1b).

### 2.3. Expression of TNF-α, IL-6 and IFN-γ in Non-Cultured CD3+ and CD3+CD56+ Cells

**Figure 2:**
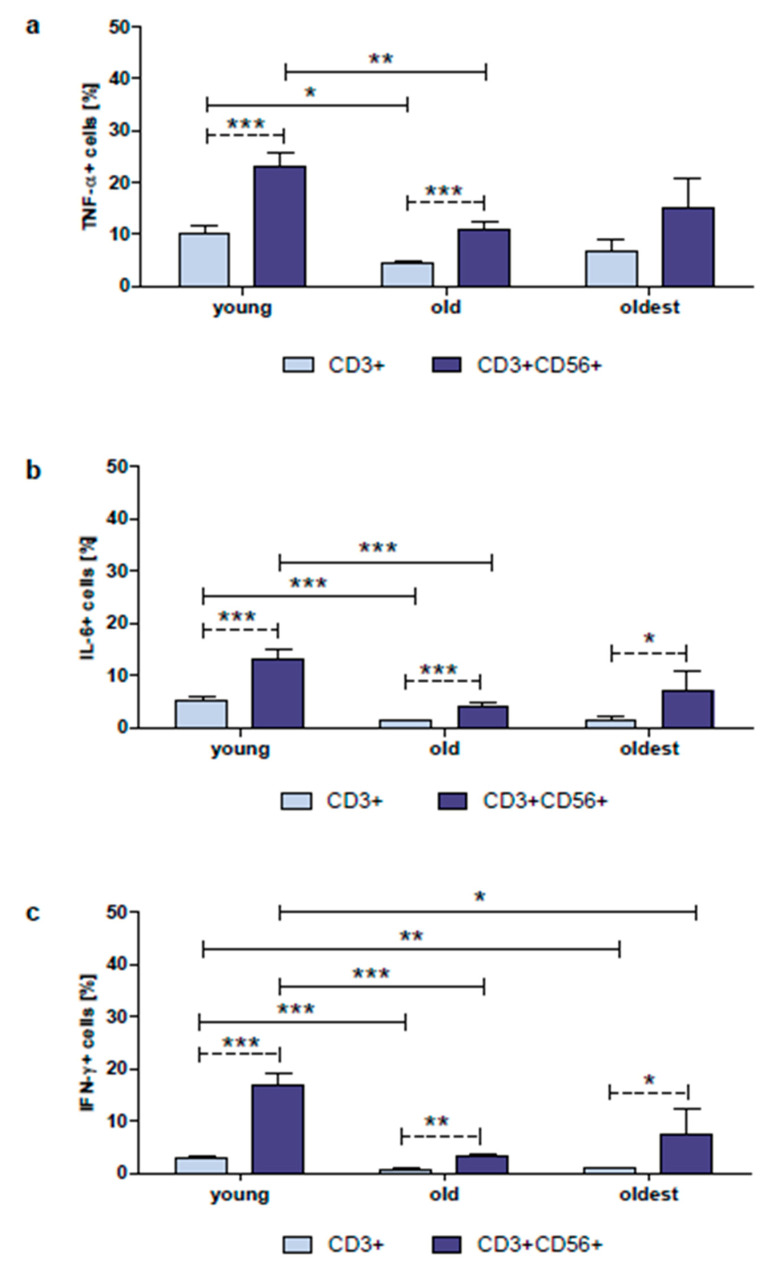
Expression of the selected cytokines in non-cultured CD3+ and CD3+CD56+ cells of the young, old, and the oldest. Data are presented as the mean ± SEM and show the intracellular expression of TNF-α, IL-6, and IFN-γ in the studied cell populations demonstrated as the percentage of cells with the expression of the particular cytokine (%). Solid horizontal lines denote statistically significant differences between the studied age groups. Dashed horizontal lines above paired bars denote statistically significant differences between CD3+ vs. CD3+CD56+ cells within the same age group. The respective symbols denote: * *p* < 0.05, ** *p* < 0.01, *** *p* < 0.001. (**a**) Expression of TNF-α in CD3+ and CD3+CD56+ cells (%). (**b**) Expression of IL-6 in CD3+ and CD3+CD56+ cells (%). (**c**) Expression of IFN-γ in CD3+ and CD3+CD56+ cells (%).

The gating strategy performed for flow cytometric analysis of T and NKT-like cells from whole blood samples is shown in Figure S1. The expression of TNF-α in non-cultured CD3+ and CD3+CD56+ cells was the highest in the young both in T and NKT-like cells and was significantly higher compared to the old in both studied subpopulations of lymphocytes. There were no statistically significant differences in TNF-α expression between the young and the oldest and both senior groups in the analyzed subpopulations of lymphocytes. Interestingly, the expression of TNF-α was over twofold higher in CD3+CD56+ cells compared to CD3+ cells in the young and the old. A similar tendency was observed in the group of the oldest, however, there was no statistical significance (Figure 2a).

When the frequency of cells expressing IL-6 was analyzed, it appeared that the percentage of cells revealing the expression of this cytokine was lower compared to TNF-α in all studied age groups, although these differences were not statistically significant. Similar to TNF-α, the expression of IL-6 in non-cultured CD3+ and CD3+CD56+ cells was the highest in the young both in T and NKT-like cells and was significantly higher compared to the old in both studied subpopulations of lymphocytes. There were no statistically significant differences in the IL-6 expression between the young and the oldest and both senior age groups in the analyzed subpopulations of lymphocytes. Furthermore, the expression of IL-6 was from 2.5 up to 4.7 times higher in CD3+CD56+ cells compared to CD3+ cells in all studied age groups (Figure 2b).

The expression of IFN-γ in non-cultured CD3+ and CD3+CD56+ cells was the highest in the young both in T and NKT-like cells and was significantly higher compared to the old and the oldest in both studied subpopulations of lymphocytes. There were no statistically significant differences in IFN-γ expression between the old and the oldest in both analyzed subpopulations of lymphocytes. Remarkably, the expression of IFN-γ was from 4.6 up to 8.9 times higher in CD3+CD56+ cells compared to CD3+ cells in all studied age groups (Figure 2c).

### 2.4. Expression of TNF-α in Cultured, Unstimulated or Stimulated with IL-2, LPS and PMA with Ionomycin CD3+ and CD3+CD56+ Cells

**Figure 3:**
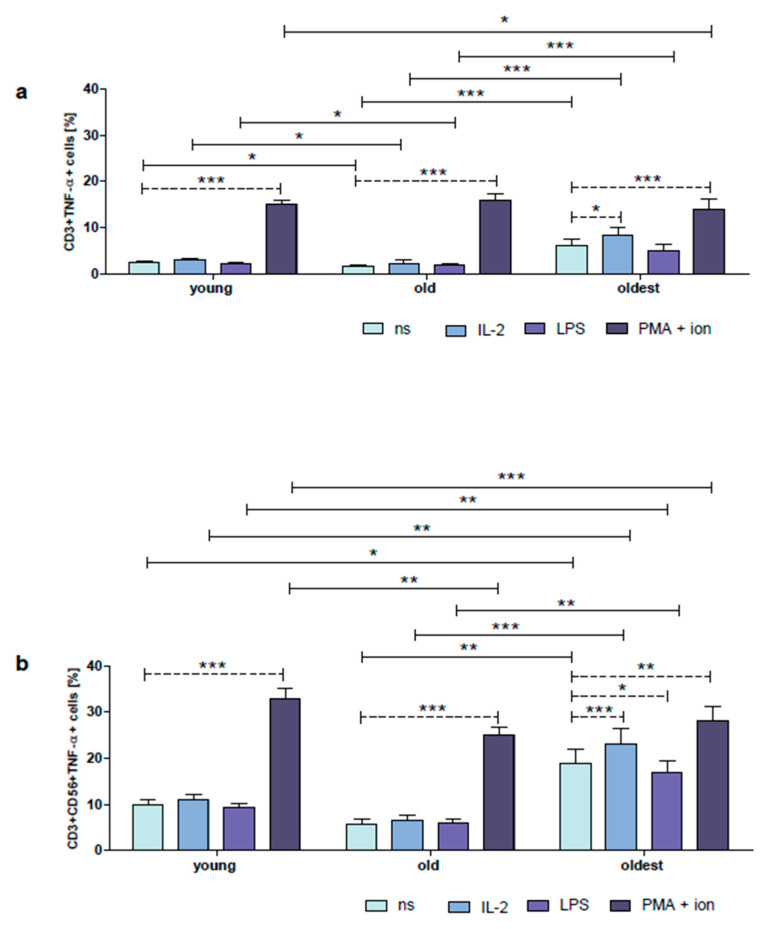
Expression of TNF-α in cultured, non-stimulated, and stimulated with IL-2, LPS, or PMA with ionomycin CD3+ and CD3+CD56+ cells of the young, old, and the oldest. Data are presented as the mean ± SEM and show the expression of TNF-α in the studied cell populations, T cells (**a**) and NKT-like cells (**b**), demonstrated as the percentage of cells with the expression of the cytokine (%). Solid horizontal lines denote statistically significant differences between similarly treated cells of different age groups, i.e., young vs. old, young vs. oldest, and old vs. oldest. Dashed horizontal lines above paired bars denote statistically significant differences between non-stimulated vs. stimulated cells within the same age group. The respective symbols denote * *p* < 0.05, ** *p* < 0.01, and *** *p* < 0.001. (**a**) Expression of TNF-α in CD3+ cells (%). (**b**) Expression of TNF-α in CD3+CD56+ cells (%).

The gating strategy performed for flow cytometric analysis of T and NKT-like cells from PBMCs cultivated 48 h in vitro is shown in Figure S2. FACS histogram overlay plots for TNF-α expression in unstimulated and stimulated CD3+ and CD3+CD56+ cells demonstrated in the representative samples of the young, old, and the oldest group are shown in Figure S3. TNF-α in unstimulated CD3+ cells of the young presented a low level of expression, i.e., 2.5 ± 0.3%, with no significant differences observed between these cells and cells stimulated with IL-2 or LPS. CD3+ cells of the young stimulated with PMA and ionomycin, however, presented a sixfold increase in TNF-α expression compared to untreated cells (Figure 3a). Interestingly, the expression of TNF-α in non-stimulated CD3+CD56+ cells of the young was fourfold higher compared to CD3+ cells and the same principle applied to the CD3+CD56+ cells stimulated with IL-2, LPS, or PMA with ionomycin which showed TNF-α expression from 2.2 up to 4.2 times higher compared to CD3+ cells (*p* < 0.0001) (Figure 3a,b). Similar to CD3+ cells, there were no significant differences in TNF-α expression observed between non-stimulated and stimulated with IL-2 and LPS NKT-like cells. However, CD3+CD56+ cells of the young stimulated with PMA and ionomycin presented TNF-α expression over threefold higher than untreated cells (Figure 3b).

The percentage of unstimulated CD3+ cells expressing TNF-α was significantly lower in the group of old compared to the young (1.7 ± 0.4%). Similar to the young, there were no significant differences between these cells and cells stimulated with IL-2 or LPS. However, CD3+ cells of the old stimulated with PMA and ionomycin showed an over ninefold increase in TNF-α expression compared to untreated cells (Figure 3a). Remarkably, the expression of TNF-α in unstimulated CD3+CD56+ cells of the old was over threefold higher than in CD3+ cells. The same observation concerned CD3+CD56+ cells stimulated with IL-2, LPS, or PMA with ionomycin which presented TNF-α expression from 1.6 up to 3.3 times higher compared to CD3+ cells (*p* < 0.0001) (Figure 3a,b). Similar to CD3+ cells, there were no significant differences in TNF-α expression observed between unstimulated and stimulated with IL-2 and LPS CD3+CD56+ cells. However, NKT-like cells of the old stimulated with PMA and ionomycin showed TNF-α expression over fourfold higher compared to unstimulated cells (Figure 3b).

The highest expression of TNF-α in non-stimulated CD3+ cells was found in the group of the oldest seniors (6.1 ± 1.4%). Interestingly, the T lymphocytes of the oldest showed also the highest sensitivity to stimulation. A significant increase in TNF-α expression was observed in CD3+ cells stimulated with IL-2 (1.3-fold increase) and PMA with ionomycin (2.3-fold increase) compared to untreated cells (Figure 3a). Intriguingly, CD3+CD56+ cells of the oldest also presented the highest expression of TNF-α in non-stimulated cells compared to the old and the young (19.0 ± 3%) and the highest sensitivity to stimulation. Similar to CD3+ cells, in CD3+CD56+ cells, a significant increase in TNF-α expression was observed for IL-2 (1.2-fold increase) and PMA with ionomycin treatment (1.5-fold increase) when compared to untreated cells. However, after stimulation with LPS, there was a significant decrease in expression of this cytokine; i.e., 17.0 ± 2.5% (Figure 3b). Moreover, the expression of TNF-α in non-stimulated CD3+CD56+ cells was over threefold higher than in CD3+ cells. The same principle concerned CD3+CD56+ cells stimulated with IL-2, LPS, or PMA with ionomycin which revealed 2–3 fold higher TNF-α expression compared to similarly treated CD3+ cells (*p* < 0.0001) (Figure 3a,b).

### 2.5. Expression of IL-6 in Cultured, Unstimulated or Stimulated with IL-2, LPS and PMA with Ionomycin CD3+ and CD3+CD56+ Cells

**Figure 4:**
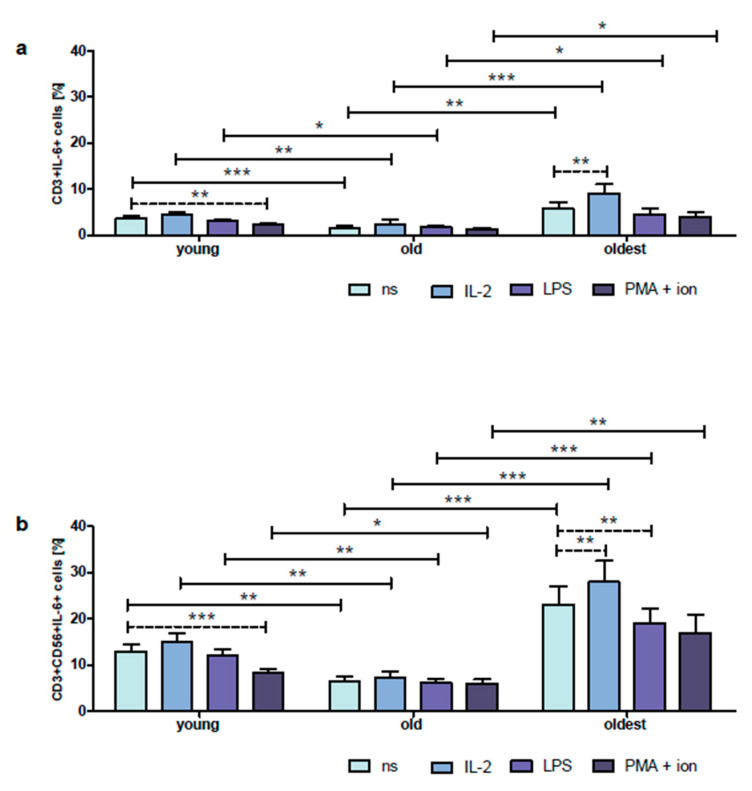
Expression of IL-6 in cultured, non-stimulated, and stimulated with IL-2, LPS, or PMA with ionomycin CD3+ and CD3+CD56+ cells of the young, old, and the oldest. Data are presented as the mean ± SEM and show the expression of IL-6 in the studied cell populations, T cells (**a**) and NKT-like cells (**b**), demonstrated as the percentage of cells with the expression of the cytokine (%). Solid horizontal lines denote statistically significant differences between similarly treated cells of different age groups, i.e., young vs. old, young vs. oldest, and old vs. oldest. Dashed horizontal lines above paired bars denote statistically significant differences between non-stimulated vs. stimulated cells within the same age group. The respective symbols denote * *p* < 0.05, ** *p* < 0.01, and *** *p* < 0.001. (**a**) Expression of IL-6 in CD3+ cells (%). (**b**). Expression of IL-6 in CD3+CD56+ cells (%).

FACS histogram overlay plots for IL-6 expression in unstimulated and stimulated CD3+ and CD3+CD56+ cells demonstrated in the representative samples of the young, old, and oldest are shown in Figure S3. IL-6 in non-stimulated CD3+ cells of the young showed slightly higher expression than TNF-α, i.e., 3.6 ± 0.5%; however, this difference was not statistically significant. Similar to TNF-α, no significant differences were found between these control, unstimulated cells and CD3+ cells treated with IL-2 or LPS. Remarkably, CD3+ cells stimulated with PMA and ionomycin in the group of the young presented a significant 1.6-fold decrease in IL-6 expression compared to untreated cells (Figure 4a). Then, the percentage of IL-6 expressing CD3+CD56+ cells within the population of untreated NKT-like cells of the young was 3.6-fold higher compared to CD3+ cells. The same observation concerned CD3+CD56+ lymphocytes stimulated with IL-2, LPS, or PMA with ionomycin which revealed IL-6 expression from 3.4 up to 4 times higher than in CD3+ cells (*p* < 0.0001) (Figure 4a,b). Likewise, there were no significant differences between unstimulated and stimulated with IL-2 or LPS CD3+CD56+ cells. Moreover, correspondingly to CD3+ cells, NKT-like cells of the young treated with PMA and ionomycin showed IL-6 expression 1.5-fold lower than non-stimulated cells (*p* < 0.001) (Figure 4b).

The percentage of non-stimulated CD3+ cells expressing IL-6 was significantly lower in the group of the old (1.6 ± 0.3%). Interestingly, there were no significant differences between unstimulated and stimulated with IL-2, LPS, or PMA with ionomycin cells in this age group (Figure 4a). Similar to TNF-α, the expression of IL-6 in untreated CD3+CD56+ cells of the old was fourfold higher than in CD3+ cells. The same principle concerned CD3+CD56+ cells stimulated with IL-2, LPS, or PMA with ionomycin which showed IL-6 expression from 3.0 up to 4.5 times higher compared to CD3+ cells (*p* < 0.0001) (Figure 4a,b). Similar to CD3+ cells of the old, there were no significant differences observed between unstimulated and stimulated with IL-2, LPS, or PMA with ionomycin CD3+CD56+ cells (Figure 4b).

The highest expression of IL-6 in unstimulated CD3+ cells was found in the group of the oldest seniors (5.7 ±1.4%). Remarkably, T cells of the oldest were sensitive to stimulation with IL-2 which resulted in 1.6-fold significant increase in the expression of this cytokine. However, there were no significant differences between untreated and treated with LPS or PMA with ionomycin CD3+ cells (Figure 4a). Noteworthy, CD3+CD56+ cells of the oldest also showed the highest expression of IL-6 in non-stimulated cells, fourfold higher than in CD3+ cells (23 ± 4%), and the same principle concerned NKT-like cells stimulated with IL-2, LPS, or PMA with ionomycin which presented IL-6 expression from 3.1 up to 4.3 times higher than in CD3+ cells (*p* < 0.0001) (Figure 4a,b). Similar to T cells, CD3+CD56+ cells of the oldest revealed sensitivity to stimulation with IL-2 which resulted in a significant 1.2-fold increase in IL-6 expression. However, after stimulation with LPS, there was a significant 1.2-fold decrease in the expression of this cytokine. There was no difference in the percentage of IL-6 expressing cells between untreated and stimulated with PMA and ionomycin CD3+CD56+ cells in the group of the oldest seniors (Figure 4b).

### 2.6. Expression of IFN-γ in Cultured, Non-Stimulated or Stimulated with IL-2, LPS and PMA with Ionomycin CD3+ and CD3+CD56+ Cells

**Figure 5:**
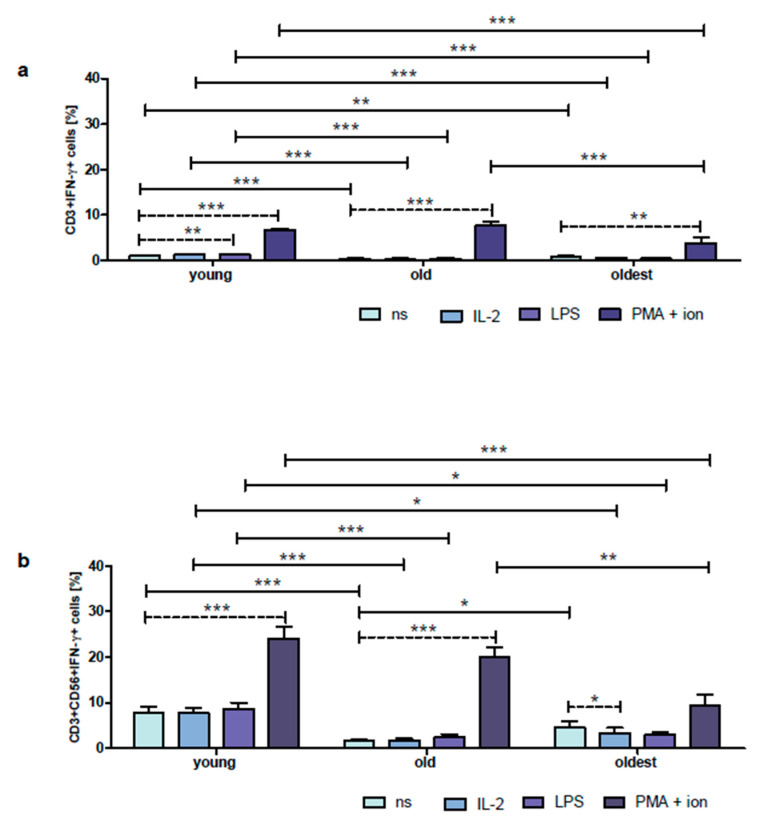
Expression of IFN-γ in cultured, non-stimulated, and stimulated with IL-2, LPS, or PMA with ionomycin CD3+ and CD3+CD56+ cells of the young, old, and oldest. Data are presented as the mean ± SEM and show the expression of IFN-γ in the studied cell populations, T cells (**a**) and NKT-like cells (**b**), demonstrated as the percentage of cells with the expression of the cytokine (%). Solid horizontal lines denote statistically significant differences between similarly treated cells of different age groups, i.e., young vs. old, young vs. oldest, and old vs. oldest. Dashed horizontal lines above paired bars denote statistically significant differences between non-stimulated vs. stimulated cells within the same age group. The respective symbols denote * *p* < 0.05, ** *p* < 0.01, *** *p* < 0.001. (**a**) Expression of IFN-γ in CD3+ cells (%). (**b**) Expression of IFN-γ in CD3+CD56+ cells (%).

FACS histogram overlay plots for IFN-γ expression in unstimulated and stimulated CD3+ and CD3+CD56+ cells demonstrated in the representative samples of the young, old, and oldest are shown in Figure S3. Then Figures S4–S7 present the frequency of samples differing in IFN-γ expression in unstimulated and stimulated CD3+ and CD3+CD56+ cells of the young, old, and oldest. The unstimulated CD3+ cells of the young showed low expression of IFN-γ, i.e., 1.0 ± 0.1%. There was no significant difference between the expression of this cytokine in non-stimulated and stimulated with IL-2 cells; however, a significant increase in IFN-γ expression in CD3+ cells treated with LPS (1.3 ± 0.1%) and PMA with ionomycin (6.6 ± 0.4%) was found (Figure 5a). Interestingly, the percentage of IFN-γ expressing cells within the population of non-stimulated CD3+CD56+ cells of the young was 7.8 times higher compared to CD3+ cells and the similar principle applied to NKT-like cells stimulated with IL-2, LPS, or PMA with ionomycin which showed IFN-γ expression from 3.6 up to 6.6 times higher than in CD3+ cells (*p* < 0.0001) (Figure 5a,b). Noteworthy, there were no significant differences between non-stimulated CD3+CD56+ cells of the young and cells stimulated with IL-2 or LPS in contrast to NKT-like cells treated with PMA and ionomycin which revealed over threefold increase in IFN-γ expression (Figure 5b).

The percentage of non-stimulated CD3+ cells expressing IFN-γ was significantly lower in the old (0.4 ± 0.1%). Remarkably, there were no differences between the expression of this cytokine in untreated T cells and cells stimulated with IL-2 or LPS. However, the stimulation with PMA and ionomycin resulted in a nineteen-fold increase in IFN-γ expression (Figure 5a). Then, the expression of IFN-γ in unstimulated CD3+CD56+ cells of the old was fourfold higher than in CD3+ cells. Likewise, NKT-like cells stimulated with IL-2, LPS or PMA with ionomycin revealed IFN-γ expression from 2.6 up to 6.3 times higher compared to T cells (*p* < 0.0001) (Figure 5a,b). Similar to CD3+ cells, there were no differences in IFN-γ expression between unstimulated and stimulated with IL-2 or LPS cells on the contrary to CD3+CD56+ cells stimulated with PMA and ionomycin which presented 12.5-fold increase of IFN-γ expression (Figure 5b).

The expression of IFN-γ in unstimulated T cells of the oldest was lower than in the young; however, it did not differ significantly from T lymphocytes of the old (0.8 ± 0.3%). Similar to the old, there were no differences between the untreated and stimulated with IL-2 or LPS cells. The stimulation with PMA and ionomycin resulted in a nearly fivefold increase in IFN-γ expression (Figure 5a). Remarkably, the non-stimulated CD3+CD56+ cells presented a 5.6-fold increase in expression of IFN-γ compared to CD3+ cells and a similar principle applied to the cells stimulated with IL-2, LPS, or PMA with ionomycin which showed IFN-γ expression from 2.5 up to 6.4 times higher than in CD3+ cells (*p* < 0.0001) (Figure 5a,b). Interestingly, NKT-like cells of the oldest were not sensitive to stimulation with LPS or PMA and ionomycin. Moreover, the stimulation of CD3+CD56+ cells with IL-2 resulted in a significant decrease in the expression of IFN-γ (3.4 ± 1.1%) (Figure 5b).

### 2.7. Relationships between Age and Serum Concentrations of α-Klotho and -SH Groups

Analysis of correlations in the studied population revealed a moderate positive correlation between α-Klotho and -SH group concentration in serum (Rs = 0.41; *p* < 0.001), a moderate negative correlation between α-Klotho serum concentration and age (Rs = −0.68; *p* < 0.001), and a moderate negative correlation between -SH groups serum concentration and age (Rs = −0.54; *p* < 0.001).

### 2.8. Relationships between the Studied Parameters Analyzed in Non-Cultured CD3+ and CD3+CD56+ Cells

Correlation analysis performed for non-cultured T and NKT-like cells revealed some relationships between the expression of the analyzed cytokines and the studied parameters. The results are presented in Table 1. Interestingly, the expression level of all studied cytokines showed a weak up to moderate positive correlation with the concentration of -SH groups in serum both for CD3+ and CD3+CD56+ cells. IL-6 and IFN-γ presented also a weak up to moderate negative correlation with age in the studied cell populations. Intriguingly, only IFN-γ revealed a weak; however, statistically significant positive correlation with α-Klotho protein serum concentration both in CD3+ and CD3+CD56+ cells (Table 1).

### 2.9. Relationships between the Studied Parameters Analyzed in Cultivated CD3+ and CD3+CD56+ Cells

Correlation analysis performed for CD3+ and CD3+CD56+ cells cultivated for 48 h concerned both non-stimulated cells and cells stimulated with IL-2, LPS, or PMA with ionomycin as described in Materials and Methods. Interestingly, the IFN-γ expression revealed a statistically significant weak up to moderate negative correlation with age for both unstimulated and stimulated T and NKT-like cells. Then, TNF-α expression in CD3+ cells presented a weak positive correlation with age; however, only for cells stimulated with IL-2 or LPS but not with PMA and ionomycin.

IFN-γ expression in T cells showed a weak, however, statistically significant positive correlation with the level of -SH groups in serum for both non-stimulated and stimulated with IL-2 or LPS cells. IFN-γ expression in NKT-like cells revealed only a weak positive correlation with cells treated with IL-2.

The most interesting relationships concerned IFN-γ expression in both CD3+ and CD3+CD56+ cells and α-Klotho serum concentration. Both populations of lymphocytes showed weak up to moderate significant positive correlations between α-Klotho concentration and IFN-γ expression which regarded both unstimulated cells and cells treated with all applied stimulating factors (Table 2).

## 3. Discussion

Aging of the immune system is characterized both by increased indices of inflammation [11] and decreased immune response that result from changes ongoing in innate and adaptive immunity [53,54]. Although during aging immune homeostasis is disturbed, these changes are not always deleterious but may be also adaptive and involved in the remodeling of the immune system [55]. We think that the presented study provides some new knowledge concerning alterations of the immune system activity and involvement of the anti-inflammatory α-Klotho protein in the maintenance of immune homeostasis in the process of aging that is usually accompanied by a pro-inflammatory state.

Klotho, a protein revealing anti-inflammatory and anti-oxidative effects [17], is thought to be associated with healthy aging and longevity by inhibition of the insulin/IGF-1 signaling pathway which negatively regulates FOXO transcription factors involved in the upregulation of numerous homeostatic genes, including catalase and mitochondrial manganese superoxide dismutase [56,57]. Our data as well as those previously reported by Yamazaki et al. and Pedersen et al. [58,59] indicated a decline of α-Klotho serum concentration with aging. This phenomenon was accompanied by a prominent decrease in the content of -SH groups in serum, although significant differences were not observed between the two groups of seniors. The concentration of sulfhydryl groups reflects the level of oxidative stress which increases with age and decreases serum concentration of thiol groups, shifting the thiol/disulfide redox balance to the more oxidative state [60]. This phenomenon characterizes the process of aging and has been reported by other authors [61] as well as by our group in earlier studies [62].

Our study compared further the effects of aging on the expression of pro-inflammatory cytokines, i.e., TNF-α or IL-6, and IFN-γ, revealing both pro-inflammatory and anti-inflammatory properties in non-cultured T and NKT-like cells and cells cultured for 48 h in the absence (non-stimulated, control cells) or presence of stimulating agents, i.e., IL-2, LPS or PMA with ionomycin. This comprehensive approach comparing the secretory activity of T and NKT-like cells in the process of aging is a new value, as to our knowledge, there are no similar studies on NKT-like cells in the context of human healthy aging. We showed that in all studied age groups non-cultured CD3+CD56+ cells revealed higher expression of TNF-α, IL-6, and IFN-γ than CD3+ cells and these observations concerned all analyzed cytokines. This phenomenon might result from the earlier observation described by Guia et al. that in contrast to CD56- T cells, CD56+ T cells were able to produce IFN-γ without earlier stimulation of TCR revealing NK-like effector functions [63]. Then, the observed expression of cytokines in non-cultured cells may result from blood sample collection and subsequent procedures which can disrupt cellular homeostasis and activate signaling pathways, increasing the expression of genes coding for some cellular protective proteins [64] and also cytokines [65].

Moreover, both T and NKT-like cells revealed the highest secretory activity in the young when compared with both senior groups. Likewise, when NK cell activity was analyzed in our previous studies [66], we found the highest expression of both TNF-α and IFN-γ in NK cells of the young, similar to NKT-like cells in the presented study. There are no comparable studies concerning differences in basal cytokine secretion between T and NKT-like cells in the process of aging. However, Schindowski et al. found in T cells higher intracellular expression of both TNF-α (significant in CD4+ cells and non-significant in CD8+ cells) and IFN-γ (non-significant) in the young compared to the old estimated by flow cytometry analysis [67]. In contrast, Hassouneh et al. showed in studies performed also by flow cytometry method that in CD4+ T cells significantly higher IFN-γ expression was found in CMV seropositive old individuals compared to middle-aged ones and a similar tendency was found for seronegative subjects. Furthermore, TNF-α expression in CD4+ T cells presented an analogous tendency in seropositive individuals. In CD8+ T cells, this tendency was observed for the expression of TNF-α both in seronegative and seropositive subjects and for IFN-γ expression only in seronegative ones. When NKT-like cells were analyzed in both CMV seronegative and seropositive individuals, a comparable tendency was observed; however, statistically significant differences between middle-aged and old persons were not found for neither TNF-α nor for IFN-γ. The authors found also that CMV infection influenced the activity of T cells and caused a functional shift associated with CMV seropositivity that differed among T cell subsets [68].

Furthermore, the introduction of IL-6 to the panel of the studied intracellular cytokines analyzed both in T and NKT-like cells adds to the novelty of the study as IL-6 is the cytokine produced mainly by monocytes and macrophages and only to some extent by other cell types, including T and NK cells [44,45]. Thus, the intracellular expression of this cytokine is usually not analyzed in T or NKT-like cells, although its serum concentration is a common parameter used in studies on the process of aging which reveals increasing concentration with age [62,69]. Our studies showed, however, that there is also a basal expression of IL-6 in non-cultured T and NKT-like cells.

Then, the analysis of the effects of stimulation with IL-2, LPS, and PMA with ionomycin of both studied lymphocyte populations in various age groups was performed. The rationale for the choice of the stimulating agents was their involvement in various signaling pathways initiating the process of lymphocyte activation. IL-2 is a major growth factor for T and NK cells [70] associated with their activation [71], resulting in the synthesis of pro-inflammatory (IL-4) and both pro- and anti-inflammatory (IFN-γ) cytokines in NKT cells [72]. Lipopolysaccharide (LPS) is a component of the outer membrane of Gram-negative bacteria and activates cells of both innate [73] and adaptive immunity [74]. Phorbol 12-myristate 13-acetate (PMA) is a protein kinase C (PKC) activator applied for strong and nonspecific stimulation of both T [75,76] and NK cells [76,77]. It is used in combination with ionomycin, a calcium ion channel-opening antibiotic that mimics the action of IP3 (inositol triphosphate), increasing the Ca2+ concentration in the cytoplasm [78]. The study of these various stimulants seemed to be interesting also regarding α-Klotho protein revealing both anti-oxidative and anti-inflammatory properties [22,23] which was also found to be involved in mechanisms of aging [13] and multiple signaling pathways [14,21].

The comparative analysis of the influence of stimulation with various stimulating agents on cytokine secretion in T and NKT-like cells provided also some new data, especially regarding NKT-like cells and the comparison between NKT-like and T cells in the process of human aging. Interestingly, the most sensitive to the process of stimulation were both CD3+ and CD3+CD56+ cells of the oldest seniors regarding TNF-α expression. On the contrary, when the expression of IFN-γ was evaluated, the most sensitive to stimulation appeared to be CD3+ cells of the young.

The presented data correspond also to our studies on cellular protective proteins in human NK cells and T or NKT-like cells which showed the highest expression of SIRT1 and HSP70 in both NK cells [79] and T or NKT-like cells [64] of the oldest seniors. These results indicate that the group of the oldest seniors might develop an adaptive stress response in NK, NKT-like, and T cells observed especially when the cells were cultivated in vitro. These data corresponded also to the increased sensitivity to the stimulation process observed for both SOD2 expression in our earlier study [79] and TNF-α and IL-6 cytokines in the present study.

There are no similar studies regarding the sensitivity to stimulation with various stimulating agents in different age groups to compare to our data. Van Epps et al. described the analysis of TNF-α and IFN-γ expression in the subpopulations of CD4+ and CD8+ T lymphocytes after stimulation with superantigen staphylococcal enterotoxin B (SEB) in older and younger adults. The stimulation procedure differed from ours and those studies were focused on various subpopulations of T cells; however, the authors found that CD4+ memory effector cells revealed a tendency to higher expression of IFN-γ in younger individuals compared to older ones similar to our data concerning T cells that showed statistically significant differences between young and old. In contrast, TNF-α expression was significantly higher in CD4+ effector cells of older individuals. A similar tendency was found for TNF-α expression in CD8+ cells [80] and T cells in the present study, especially regarding the comparison between the oldest seniors with the old or the young. Van der Geest et al. studied TNF-α and IFN-γ expression after stimulation with PMA and calcium ionophore in the presence of brefeldin A in CD4+ and CD8+ cells differing in the expression of CD161 antigen. These authors found significantly higher IFN-γ expression in CD4+ CD161+ cells in the group of the young compared to the old, and those results were in line with our data concerning T cells, in contrast to CD8+ cells revealing significantly higher expression of IFN-γ in CD8+ CD161- cells in the group of the old. Then CD8+ CD161- cells presented significantly higher expression of TNF-α in the group of the old and a similar tendency was observed in CD4+ CD161- cells [81]. Those results were also in line with our data concerning the expression of TNF-α in T cells of the oldest seniors vs. the young and the old.

The analysis of IL-6 expression in T and NKT-like cells performed in the studied age groups revealed that the most sensitive to stimulation were both T and NKT-like cells of the oldest. Moreover, the expression of IL-6 in T cells was significantly higher in the group of the oldest, compared to the old, and a similar tendency was observed regarding the young group. Those experiments presented a novel aspect as an intracellular expression of IL-6 has not been usually analyzed in stimulated T or NKT-like cells. Moreover, there are no studies presenting alterations in the expression of this cytokine in the process of healthy aging. There are only a few studies regarding IL-6 expression which involved the detection of this cytokine in supernatants of cell cultures isolated from healthy or unhealthy individuals. They concerned PBMC cells stimulated with αGalCer (α-Galactosylceramide) or *Eh*LPPG (*Entamoeba histolytica* lipopeptidephosphoglycan), molecules responsible for the activation of invariant NKT cells in CD1d-dependent manner. The presence of IL-6 was detected in supernatants of PBMCs isolated from young and middle-aged individuals [82]. The level of IL-6 was also analyzed with in-house-developed bead sets in supernatants of CD56+ cells (NK/NKT-like cells) isolated from PBMCs fraction of healthy controls (mean age 47.8 ± 8.4) and SSc (systemic sclerosis) patients (mean age 56.2 ± 11.3) after stimulation with TLR1/2 (Toll-like receptor) agonists and additionally with IL-2 and IFN-α. The higher production of IL-6 was observed in SSc patients in contrast to healthy controls. The authors, however, were not able to differentiate between NK and NKT-like cells [83].

Remarkably, TNF-α, IL-6 or IFN-γ expressions in cultured, stimulated, or non-stimulated CD3+CD56+ cells were a few times higher than in CD3+ cells. The relationship between CD56 expression and the state of cell activation was observed earlier by Michel et al. [84]. The authors studied CD56-expressing T cells from patients with extraarticular rheumatoid arthritis (mean age 63.9 ± 11.5) and found that CD56 molecules appeared to be functionally competent receptors on the surface of T cells which could act both as independent stimulatory receptors or as costimulatory receptors. They showed that CD56 ligation was sufficient to induce secretion of IL-2, TNF-α, and MIP-1β; however, the colligation of CD56 and the TCR-CD3 complex increased the secretion levels of these cytokines [84]. Then, Guia et al. noted that NK-like CD56+ T cells analyzed by flow cytometry revealed higher production of IFN-γ than CD56- T cells after stimulation of PBMCs from healthy blood donors with BCG alone or with BCG and IL-12 [63]. Further, NKT-like cells were described to be strong producers of pro-inflammatory cytokines; i.e., TNF-α or IL-1β, and IFN-γ, revealing both pro- and anti-inflammatory properties after the triggering of KIR2DL2/3, KIR2DL4, and NKG2A receptors in IL-15 induced CD8+NKR+ T cells analyzed by flow cytometry using fluorescent bead immunoassay [85]. Similarly, Takayama et al., showed that after stimulation of NK type (CD56+) T cells with IL-2, IL-12, and IL-15 they produced larger amounts of IFN-γ than regular CD8+ T cells. IFN-γ production was analyzed by ELISA in supernatants of CD56+ T cell subset cultures of PBMCs purified by cell sorting [86]. Those results, partly obtained with different methods, were consistent with our data concerning the expression of TNF-α and IFN-γ. The novelty of our studies concerned the comparison of the sensitivity to stimulation of the analyzed CD3+ and CD3+CD56+ cells in the process of aging.

Some interesting data resulted also from the analysis of correlations performed for both non-cultured and cultivated for 48 h cells. Interestingly, the expression of all studied cytokines in non-cultured CD3+ and CD3+CD56+ cells appeared to correlate positively with the level of oxidative stress represented by the content of –SH groups in the serum. A higher concentration of sulfhydryl groups corresponds to a lower level of oxidative stress as the conditions promoting oxidative stress result in the oxidation of free sulfhydryl groups to their corresponding disulfides [87]. Our results showed that attenuated oxidative stress reflected by a higher content of –SH groups in serum corresponded to the higher expression level of the studied cytokines; i.e., TNF-α, IL-6, and IFN-γ. A similar association between the expression of IFN-γ by NK cells and the content of –SH groups in serum was described by our group earlier [62]. Moreover, the procedure of blood sample collection may result in the activation of various stress signaling pathways promoting the expression of cytokines [65,88]. Additionally, IL-6 and IFN-γ correlated negatively with age that was also shown to negatively correlate with the content of –SH groups reflecting the level of oxidative stress [87].

An intriguing association was observed between serum Klotho concentration and expression of IFN-γ in non-cultured CD3+ and CD3+CD56+ cells. This relationship was also found in cultured cells, both unstimulated and stimulated with IL-2, LPS, or PMA with ionomycin. This positive correlation was even more interesting as it did not concern other types of the analyzed cytokines; i.e., TNF-α or IL-6. Additionally, IFN-γ is considered to serve as a marker of cell activation in response to the stimulation of T, NKT, and NK cells [89,90]. Thus, α-Klotho seems to positively correlate with the state of cellular activity and this finding adds novelty to the study as to our knowledge such an association has not yet been reported.

The percentage of IFN-γ expressing cells correlated positively with -SH group serum concentration in both unstimulated and stimulated with IL-2 or LPS CD3+ cells and stimulated with IL-2 CD3+CD56+ cells. Then, the expression of IFN-γ in both CD3+ and CD3+CD56+ cells unstimulated or treated with all applied stimulatory factors presented a weak or medium negative correlation with age. Thus, IFN-γ expression seems to correlate with the level of oxidative stress which also corresponds to age [2,3]. A similar, positive relationship between the content of -SH groups and IFN-γ was observed by our group earlier in NK cells [62]. The role of IFN-γ in inflammation and oxidative stress seems to be ambiguous as this cytokine may induce both of these processes [91,92] and may also reveal some protective and regulatory role [93,94].

These inconclusive activities of IFN-γ result from the fact that interferons can induce both oxidative stress and formation of oxidant-damaged proteins on one hand and the activity of immunoproteasomes involved in a clearance of damaged protein aggregates to preserve cell viability on the other [95,96]. Since α-Klotho is both anti-inflammatory and anti-oxidative protein, it is involved in numerous protective signaling pathways, including MAPK/ERK, SRK1/p38MAPK, cAMP/PKA, Nrf1/2, and insulin/IGF1/PI3K [14]. Thus, a significant positive correlation between the expression of IFN-γ, a cytokine corresponding to the state of cellular activity [89,90] and a protective α-Klotho protein might result from the established immune system homeostasis maintained with the involvement of IFN-γ [93]. Moreover, this association may also indicate another component of the adaptive mechanisms developed during the process of aging described earlier by our group related to the expression of cellular protective proteins in NK, NKT-like, and T cells [64,66,97].

## 4. Materials and Methods

### 4.1. Participants

Eighty-six volunteers aged between 19 and 94 years (62 women and 24 men) participated in the study. The exclusion criteria included serum CRP (C-reactive protein) concentration >5 mg/L, cancer, autoimmune disease, diabetes, infection, use of immunosuppressors, glucocorticoids or nonsteroidal anti-inflammatory drugs (NSAIDs), and moderate to severe dementia assessed using the “Mini Mental State Examination” (MMSE below 23 points) [98]. The geriatric conditions of senior volunteers were also considered by applying Katz’s scale to assess “activities of daily living” (ADL), and only seniors with a score of 5–6 points were enrolled in the study [99]. Senior volunteers were recruited among inhabitants of the local senior house in the city of Gdynia (Poland), and young volunteers were students at the Medical University of Gdańsk (Poland). The participants were subdivided into 3 groups: 31 young subjects in the study referred to as ‘young’ (mean age 20.9 ± 0.3 years, range 19–24 years, 22 women and 9 men), 30 seniors aged under 85 referred to as ‘old’ (mean age 75.6 ± 0.9 years, range 65–84 years, 20 women and 10 men), and 25 seniors aged over 85 referred to as ‘oldest’ (mean age 88.5 ± 0.5 years, range 85–94 years; 20 women and 5 men). All volunteers signed informed consent forms, and the study received approval from the Ethical Committee of the Medical University of Gdańsk, Poland (225/2010). The immunological characteristics of the study population were described in our previous studies [66,97].

### 4.2. Staining of Surface and Intracellular Antigens in Whole Blood Samples for Flow Cytometry

Whole blood samples (0.1 mL) were aliquoted into flow cytometry tubes and CD3-FITC-conjugated (0.125 μg/mL; clone UCHT1) (BD Biosciences, San Jose, CA, USA) and CD56-APC-conjugated (0.6 μg/mL; clone NCAM16.2) (BD Biosciences, San Jose, CA, USA) monoclonal antibodies were added for cell surface antigen staining. After 30 min of incubation in the dark at room temperature, 2 mL of BD FACS Lysing Solution was added, and samples were incubated for subsequent 10 min in the same conditions. Then, cells were washed twice with 1 mL of BD Staining Buffer (PBS without Ca^2+^ and Mg^2+^, 1% FBS (fetal bovine serum), and 0.09% sodium azide) and resuspended in 0.25 mL of Fixation/Permeabilization Solution for 20 min at 4 °C according to the manufacturer’s protocol (BD Cytofix/Cytoperm Fixation/Permeabilization Kit). Cells were washed twice with 1 mL of BD Perm/Wash buffer and appropriate volumes of TNF-PE-Cy7- conjugated (0.125 μg/mL; clone MAb11) (BD Biosciences, San Jose, CA, USA), IL-6-PE-conjugated (0.25 μg/mL; clone MQ2-13A5) (BD Biosciences, San Jose, CA, USA), or IFN-γ-PE-conjugated (0.125 μg/mL; clone 4S.B3) (BD Biosciences, San Jose, CA, USA) monoclonal antibodies were added for staining of intracellular antigens according to the manufacturer’s instructions. After 30 min of incubation in the dark at room temperature cells were washed twice with 1 mL of BD Perm/Wash buffer and resuspended in Staining Buffer prior to flow cytometric analysis. Samples were run on a BD FACSCalibur flow cytometer equipped with argon-ion laser (488 nm) and data were analyzed with BD CellQuest Pro software (BD Biosciences, San Jose, CA, USA) after acquiring 10,000 gated events (lymphocytes). Peripheral blood lymphocytes were gated using forward (FSC) and side scatter (SSC) parameters. NKT-like cells were identified as CD3+CD56+ cells and T lymphocytes were classified as CD3+ cells. Then, CD3+ and CD3+CD56+ subsets were analyzed for the frequency of cells expressing particular intracellular cytokines; i.e., TNF-α, IL-6, and IFN-γ. Appropriate isotype controls were prepared. Staining and fixation procedures were carried out within 4 h after blood sample collection.

### 4.3. Preparation of Peripheral Blood Mononuclear Cell Cultures

Peripheral blood mononuclear cells (PBMCs) were isolated from venous blood samples collected in tubes with EDTA by conventional ficoll-uropoline density gradient centrifugation. PBMCs were then washed with phosphate-buffered saline (PBS) and resuspended in RPMI1640 medium supplemented with 5% FBS, penicillin (100 U/mL-streptomycin (100 μg/mL), and 2-mercaptoethanol (5 × 10^−5^ M) (all purchased from Sigma Aldrich, Saint Louis, MO, USA). The expression of intracellular TNF-α, IL-6, and IFN-γ was studied in PBMCs cultivated for 48 h in vitro (5 × 10^5^/0.5 mL) in the presence of IL-2 (100 U/mL) (BD Biosciences, San Jose, CA, USA), LPS (1 μg/mL) Sigma-Aldrich, Saint Louis, MO, USA), or PMA (50 ng/mL) with ionomycin (500 ng/mL) (Sigma Aldrich, Saint Louis, MO, USA). Control cells were left without stimulation. 5 h before the end Golgi Stop reagent (0.5 μL/well in 0.5 mL of medium, BD Biosciences, San Jose, CA, USA) was added to PBMC cultures to stop extracellular export of cytokines. Then, PBMCs were collected and washed with 1 mL of BD Staining Buffer. Cell viability of PBMCs was monitored with trypan blue exclusion assay.

### 4.4. Staining of Surface and Intracellular Antigens in Peripheral Blood Mononuclear Cells for Flow Cytometry

PBMCs (2.5 × 10^5^ cells) were aliquoted into flow cytometry tubes and CD3-FITC-conjugated (0.125 μg/mL; clone UCHT1) (BD Biosciences, San Jose, CA, USA) and CD56-APC-conjugated (0.6 μg/mL; clone NCAM16.2) (BD Biosciences, San Jose, CA, USA) monoclonal antibodies were added for cell surface antigen staining. After 30 min of incubation in the dark at room temperature cells were washed twice with 1 mL of BD Staining Buffer (PBS without Ca^2+^ and Mg^2+^, 1% FBS, 0.09% sodium azide) and resuspended in 0.25 mL of Fixation/Permeabilization Solution for 20 min at 4 °C following manufacturer’s protocol (BD Cytofix/Cytoperm Fixation/Permeabilization Kit). Cells were washed twice with 1 mL of BD Perm/Wash buffer and relevant volumes of TNF-PE-Cy7-conjugated (0.125 μg/mL; clone MAb11) (BD Biosciences, San Jose, CA, USA), IL-6-PE-conjugated (0.25 μg/mL; clone MQ2-13A5) (BD Biosciences, San Jose, CA, USA) or IFN-γ-PE-conjugated (0.125 μg/mL; clone 4S.B3) (BD Biosciences, San Jose, CA, USA) monoclonal antibodies were added for staining of intracellular antigens following the manufacturer’s instructions. After 30 min of incubation in the dark at room temperature cells were washed twice with 1 mL of BD Perm/Wash buffer and resuspended in Staining Buffer prior to flow cytometric analysis. Samples were run on a BD FACS Calibur flow cytometer equipped with argon-ion laser (488 nm) and data were evaluated with BD CellQuest Pro software (BD Biosciences, San Jose, CA, USA) after collecting 10,000 gated events (lymphocytes). Peripheral blood lymphocytes were gated using forward (FSC) and side scatter (SSC) parameters. NKT-like cells were identified as CD3+CD56+ cells and T lymphocytes were classified as CD3+ cells. Then, CD3+ and CD3+CD56+ subsets were analyzed for the frequency of cells expressing particular intracellular cytokines; i.e., TNF-α, IL-6 and IFN-γ. Relevant isotype controls were also applied.

### 4.5. Serum Sample Collection

Blood collected in vacutainers without anti-coagulant was left for at least 30 min at room temperature to allow the blood to clot and then within 2 h from the time of collection was centrifuged at 1500× *g* for 10 min at room temperature. The serum was recovered and placed into cryovial tubes in aliquots and stored at −80 °C until further analysis.

### 4.6. Determination of the Total –SH Group Content in Serum

The total sulfhydryl group (–SH group) concentration in serum was measured with DTNB (2.2-dithio-bis-nitrobenzoic acid) assay. A total of 200 μL of sodium phosphate buffer (10 mM) pH 8.0 was added to each well. After the addition of 30 μL of sodium dodecyl sulfate (10%) to expose hidden sulfhydryl groups of proteins, 20 μL of serum was added and vigorously mixed. Subsequently, 30 μL of DTNB (1 mM) was added into the experimental sample (prepared in duplicates) or 30 μL of sodium phosphate buffer into the reference sample. The samples were then incubated at 37 °C for 1 h and absorbance was measured at 412 nm with Varioskan^TM^ LUX multimode microplate reader (Thermo Fisher Scientific, Waltham, MS, USA). A standard curve reflecting absorbance of the increasing concentrations of a reduced form of glutathione (GSH), (Sigma Aldrich, Saint Louis, MO, USA) was prepared and used to analyze the concentration of sulfhydryl groups in the experimental samples. The stock solution of GSH was prepared immediately before running the measurement (6.1 mg in 1 mL of Milli-Q water) and then diluted in Milli-Q water to prepare increasing concentrations of GSH. The lowest concentration of reduced glutathione in the standard curve was 0.25 mmol/L, and the range of the standard curve was between 0 and 4 mmol/L. Data were presented as nmoles of –SH groups/mg of serum protein whose concentration was measured according to Bradford assay.

### 4.7. Determination of α-Klotho Concentration in Serum

The human α-Klotho ELISA assay was carried out according to the protocol delivered by the manufacturer (IBL International, Fujioka, Japan). Briefly, 100 μL of EIA buffer was pipetted into 96 wells of the microtiter plate coated with a mouse monoclonal antibody against human α-Klotho. Then, 100 μL of blank test samples, test samples, and dilutions of standards were pipetted into the appropriate wells. The pre-coated plate was incubated for 60 min at room temperature. After washing, 100 µL of labeled antibody solution was pipetted into the wells of test samples, diluted standards, and blank test samples. The pre-coated plate was incubated for 30 min at room temperature and then washed. Subsequently, 100 µL of chromogen was pipetted into the wells and the precoated plate was incubated for 30 min at room temperature in the dark. The liquid turned blue. Then, 100 µL of stop solution was added to the wells. After mixing, the liquid turned yellow. The absorbance was measured at 450 nm with Varioskan^TM^ LUX multimode microplate reader (Thermo Fisher Scientific, Waltham, MS, USA) within 30 min after the addition of the stop solution.

### 4.8. Statistics

All data are expressed as the mean ± SEM. The normality of the data distribution was analyzed by the Shapiro–Wilk test. The Kruskal–Wallis test was used to compare experimental data for nonparametric distribution in the analysis of three independent age groups. The Mann–Whitney U test for nonparametric distribution was applied to compare two independent samples, and the Wilcoxon signed-rank test for nonparametric distribution was used to compare two related samples. The Spearman correlation coefficient (Rs) was applied to present the strength of the relationship between variables (Statistica, version 13; Statsoft, Tulsa, OK, USA). Differences or correlations with *p* < 0.05 were considered statistically significant.

## 5. Conclusions

The serum concentration of α-Klotho, a protective protein revealing both anti-inflammatory and anti-oxidative properties, decreases with age. However, it presents a positive correlation with the expression of IFN-γ in both non-cultured and cultivated NKT-like and T cells. Since IFN-γ shows both pro-inflammatory and protective properties, it is engaged in the maintenance of immune homeostasis. The observed relationships may then indicate the involvement of α-Klotho and cellular IFN-γ expression in the network of adaptive mechanisms developed during the process of human aging.

## Figures and Tables

**Table 1:** Correlation analysis concerning the expression of the analyzed cytokines in non-cultured (“0”) CD3+ cells and CD3+CD56+ cells and indicated parameters characterized in the studied population. All values are presented as statistically significant Spearman’s correlation coefficients (Rs). ‘ns’ denotes statistically not significant. The respective symbols denote: * *p* < 0.05, ** *p* < 0.01, and *** *p* < 0.001.

Cell Population	CD3+ [%]			CD3+CD56+ [%]		
**Parameter**	**TNF-α “0”**	**IL-6 “0”**	**IFN-γ “0”**	**TNF-α “0”**	**IL-6 “0”**	**IFN-γ “0”**
**α-Klotho**	ns	ns	0.37 **	ns	ns	0.34 **
**SH groups**	0.29 *	0.38 **	0.4 **	0.41 **	0.45 ***	0.36 **
**Age**	ns	−0.32 *	−0.52 ***	ns	−0.31*	−0.46 ***

**Table 2:** Correlation analysis concerning the expression of the analyzed cytokines in cultivated for 48 h unstimulated or stimulated with IL-2, LPS, and PMA with ionomycin CD3+ and CD3+CD56+ cells and indicated parameters characterized in the studied population. All values are presented as statistically significant Spearman’s correlation coefficients (Rs). ‘ns’ denotes statistically not significant. The respective symbols denote: * *p* < 0.05, ** *p* < 0.01, and *** *p* < 0.001.

Cell Population		CD3+ [%]			CD3+CD56+ [%]		
Parameter	Stimulation Type	TNF-α	IL-6	IFN-γ	TNF-α	IL-6	IFN-γ
**α-Klotho**	none	ns	ns	0.34 **	ns	ns	0.25 *
	IL-2	ns	ns	0.44 ***	ns	ns	0.35 **
	LPS	ns	ns	0.49 ***	ns	ns	0.39 ***
	PMA+ion	ns	ns	0.22 *	ns	ns	0.32 **
**-SH groups**	none	ns	ns	0.3 **	ns	ns	ns
	IL-2	ns	ns	0.36 **	ns	ns	0.22 *
	LPS	ns	ns	0.34 **	ns	ns	ns
	PMA+ion	ns	ns	ns	ns	ns	ns
**Age**	none	ns	ns	−0.32 **	ns	ns	−0.24 *
	IL-2	0.23 *	ns	−0.37 ***	ns	ns	−0.26 *
	LPS	0.26 **	ns	−0.4 ***	ns	ns	−0.23 *
	PMA+ion	ns	ns	−0.33 ***	ns	ns	−0.42 ***

## Data Availability

All data presented in the study are available from the corresponding author upon reasonable request.

## Supplementary Materials

The following supporting information can be downloaded at: https://www.mdpi.com/article/10.3390/ijms24098393/s1.
