# Supplementary material for: Anti-Inflammatory Klotho Protein Serum Concentration Correlates with Interferon Gamma Expression Related to the Cellular Activity of Both NKT-like and T Cells in the Process of Human Aging
Source: Int J Mol Sci. 2023 May 7;24(9):8393. doi: 10.3390/ijms24098393 (PMC10179552; doi:10.3390/ijms24098393)
Supplement: Supplementary file 1 [file ijms-24-08393-s001.zip › ijms-2323032-supplementary.pdf]

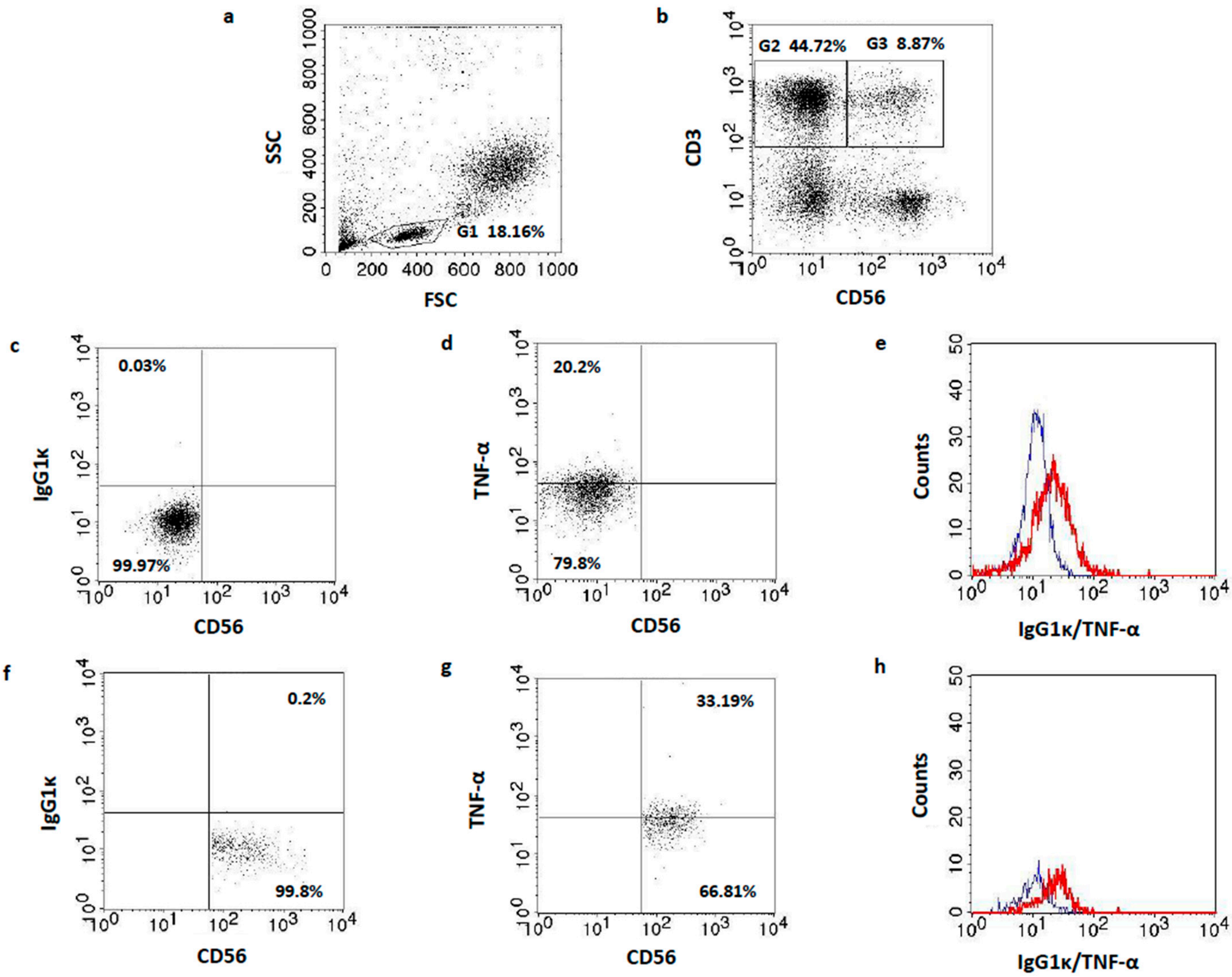

Figure S1. Gating strategy performed for flow cytometric analysis of T lymphocytes (CD3+ cells) and NKT-like cells (CD3+CD56+ cells) from whole blood samples. (**a**) Lymphocyte gating – lymphocytes were defined as FSC_{low}/SSC_{low} cells (G1). (**b**) T and NKT-like cell gating – T lymphocytes were defined as CD3+ positive population (G2), and NKT-like cells were defined as CD3+CD56+ positive cells (G3). (**c**) Isotype control for TNF-α positive T cells. (**d**) T cells expressing TNF-α were identified in the upper left quadrant. (**e**) TNF-α expression (red line) vs isotype control (blue line) presented in T cells. (**f**) Isotype control for TNF-α -positive NKT-like cells. (**g**) NKT-like cells expressing TNF-α were identified in the upper right quadrant. (**h**) TNF-α expression (red line) vs isotype control (blue line) presented in NKT-like cells. The analysis of IL-6 and IFN-γ expression was performed in the same way. All age groups were analyzed with the same gating strategy.

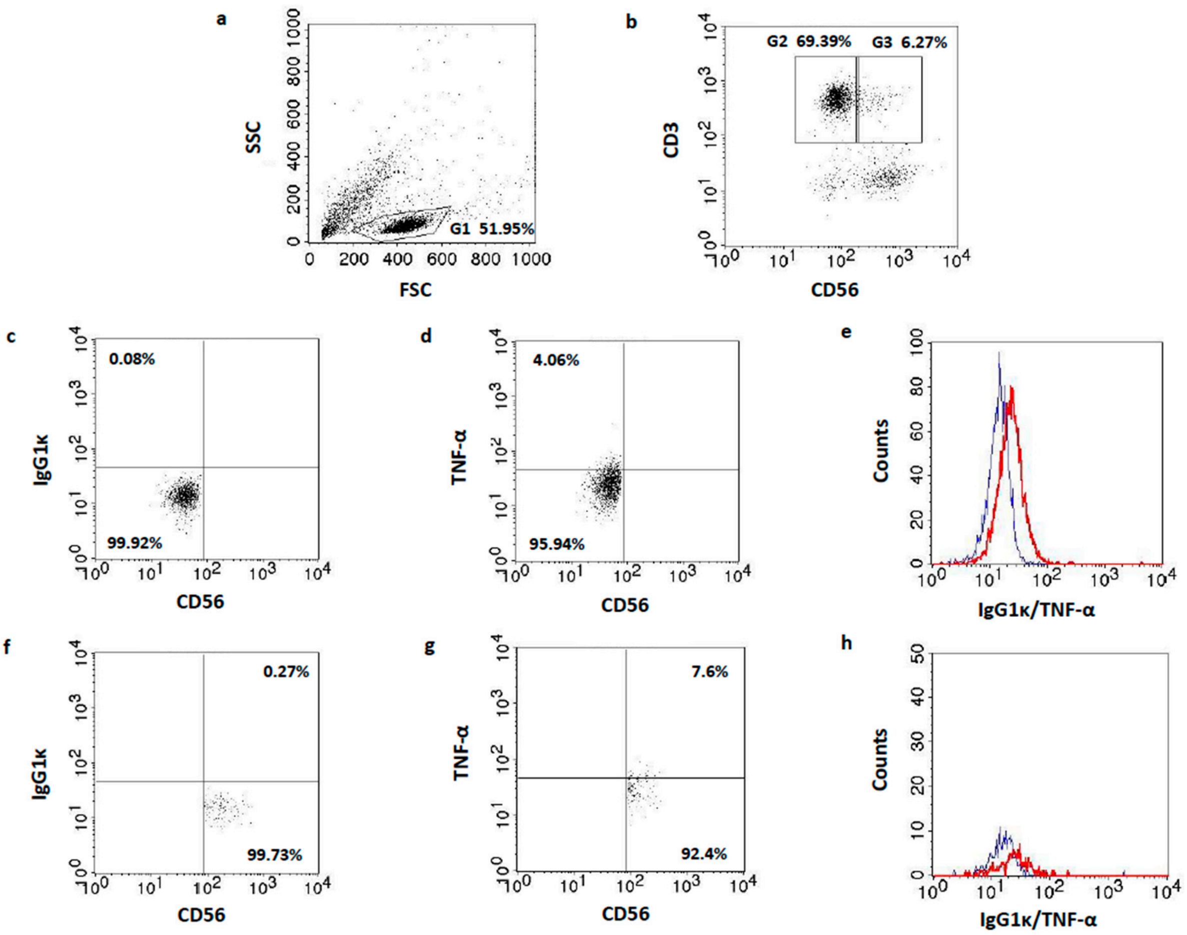

Figure S2. Gating strategy performed for flow cytometric analysis of T lymphocytes (CD3+ cells) and NKT-like cells (CD3+CD56+ cells) from PBMC cultures. (**a**) Lymphocyte gating in the population of PBMCs – lymphocytes were defined as FSC_{low}/SSC_{low} cells (G1). (**b**) T and NKT-like cell gating – T lymphocytes were defined as CD3+ positive population (G2), and NKT-like cells were defined as CD3+CD56+ positive cells (G3). (**c**) Isotype control for TNF-α -positive T cells. (**d**) T cells expressing TNF-α were identified in the upper left quadrant. (**e**) TNF-α expression (red line) vs isotype control (blue line) presented in T cells. (**f**) Isotype control for TNF-α -positive NKT-like cells. (**g**) NKT-like cells expressing TNF-α were identified in the upper right quadrant. (**h**) TNF-α expression (red line) vs isotype control (blue line) presented in NKT-like cells. The analysis of IL-6 and IFN-γ expression was performed in the same way. All age groups were analyzed with the same gating strategy.

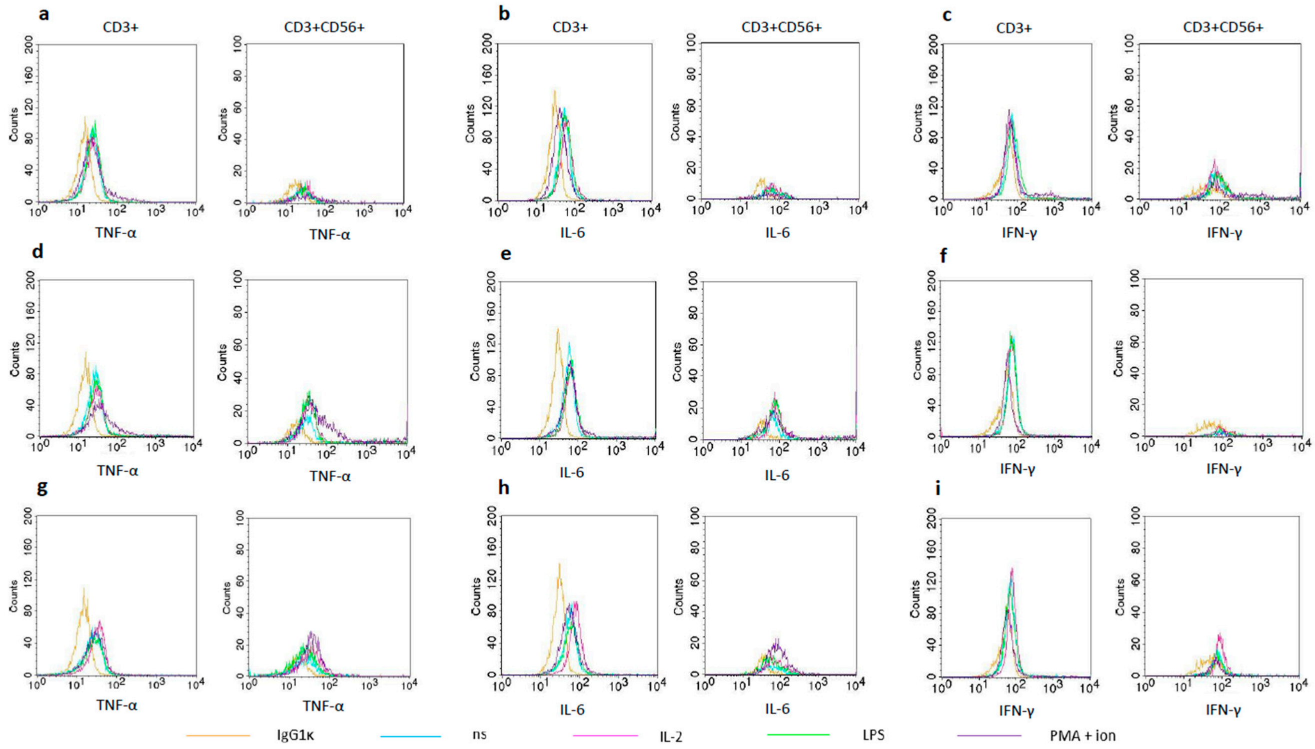

Figure S3. FACS histogram overlay plots for TNF-α, IL-6 and IFN-γ expression in CD3+ and CD3+CD56+ cells presented in the representative samples of the young, old and the oldest group. Each plot shows overlay graphs depicting isotype control (IgG1κ – orange line), unstimulated cells (ns – light blue line), cells stimulated with IL-2 (IL-2 – pink line), cells stimulated with LPS (LPS – light green line) and cells treated with PMA and ionomycin (PMA + ion – dark purple line). (**a**) TNF-α expression in CD3+ cells (left) and CD3+CD56+ cells (right) of the young. (**b**) IL-6 expression in CD3+ cells (left) and CD3+CD56+ cells (right) of the young. (**c**) IFN-γ expression in CD3+ cells (left) and CD3+CD56+ cells (right) of the young. (**d**) TNF-α expression in CD3+ cells (left) and CD3+CD56+ cells (right) of the old. (**e**) IL-6 expression in CD3+ cells (left) and CD3+CD56+ cells (right) of the old. (**f**) IFN-γ expression in CD3+ cells (left) and CD3+CD56+ cells (right) of the old. (**g**) TNF-α expression in CD3+ cells (left) and CD3+CD56+ cells (right) of the oldest. (**h**) IL-6 expression in CD3+ cells (left) and CD3+CD56+ cells (right) of the oldest. (**i**) IFN-γ expression in CD3+ cells (left) and CD3+CD56+ cells (right) of the oldest.

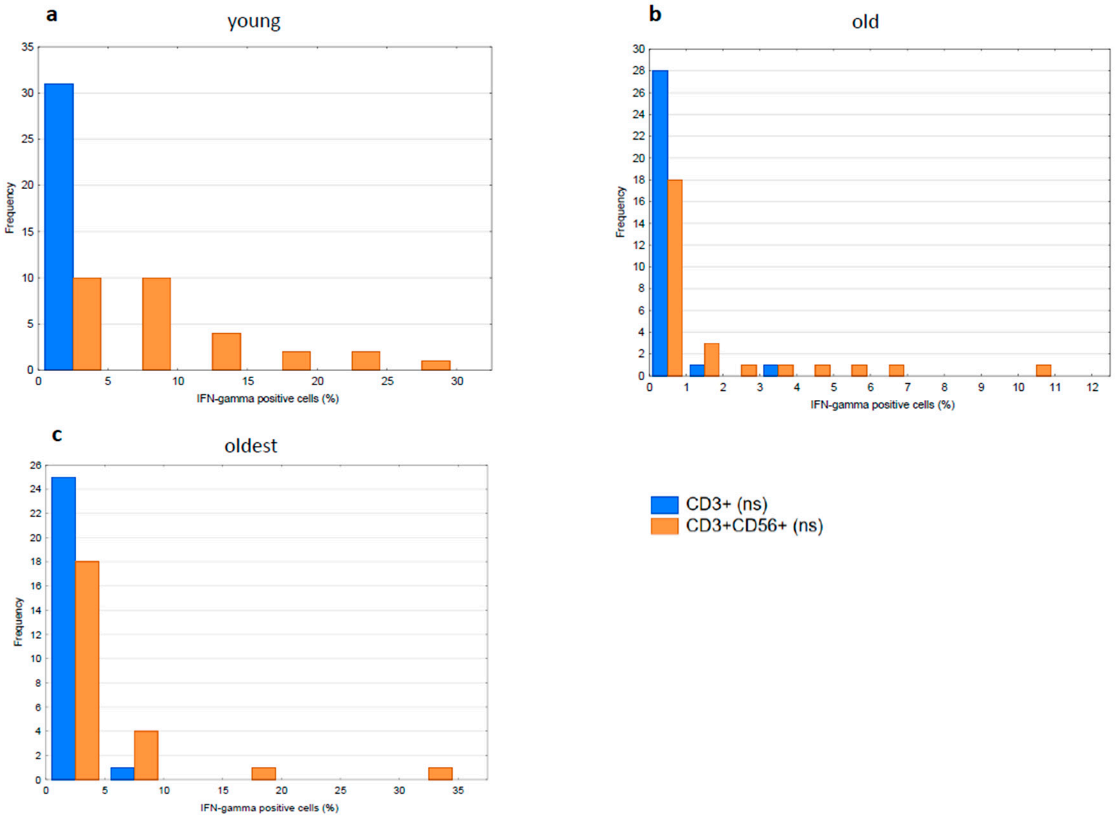

Figure S4. Frequency of samples differing in IFN-γ expression in the population of unstimulated (ns) CD3+ cells (blue color) and CD3+CD56+ cells (orange color) analysed in the group of the young (**a**), old (**b**) and the oldest (**c**).

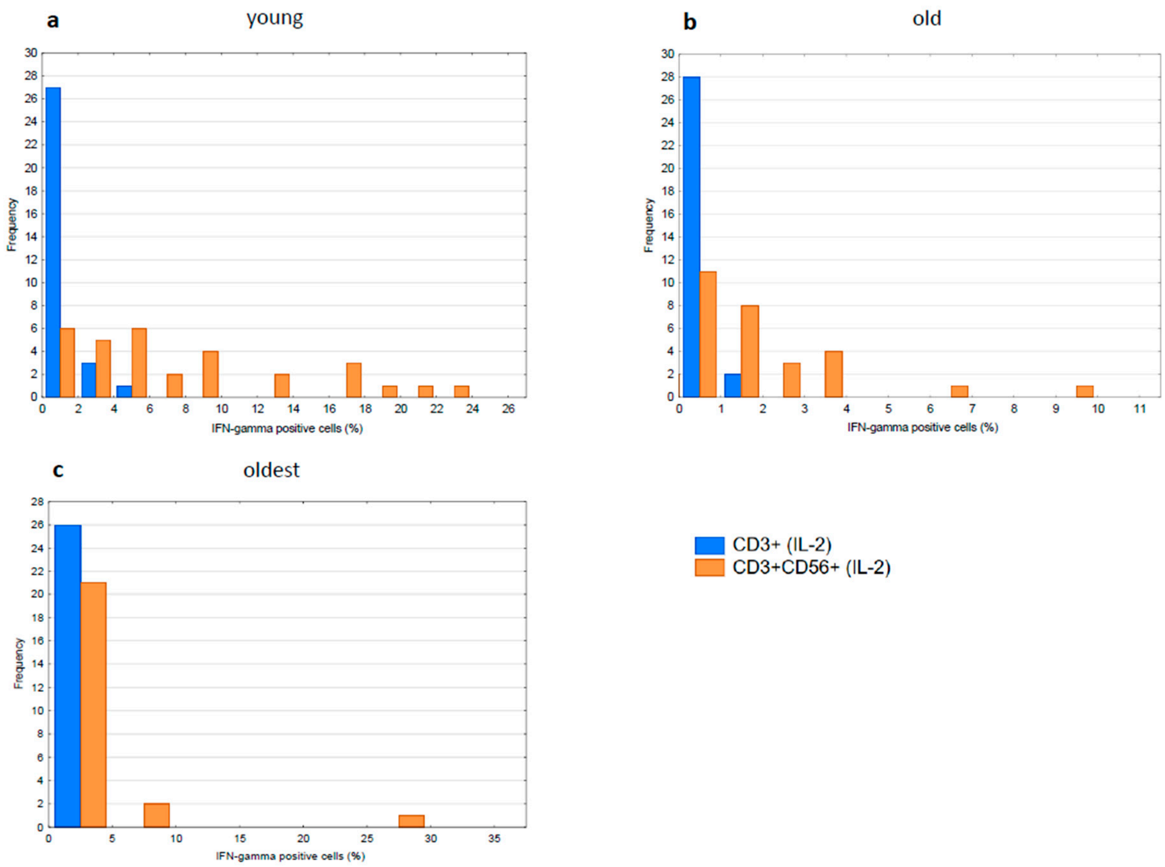

Figure S5. Frequency of samples differing in IFN-γ expression in the population of CD3+ cells (blue color) and CD3+CD56+ cells (orange color) stimulated with IL-2 analysed in the group of the young (**a**), old (**b**) and the oldest (**c**).

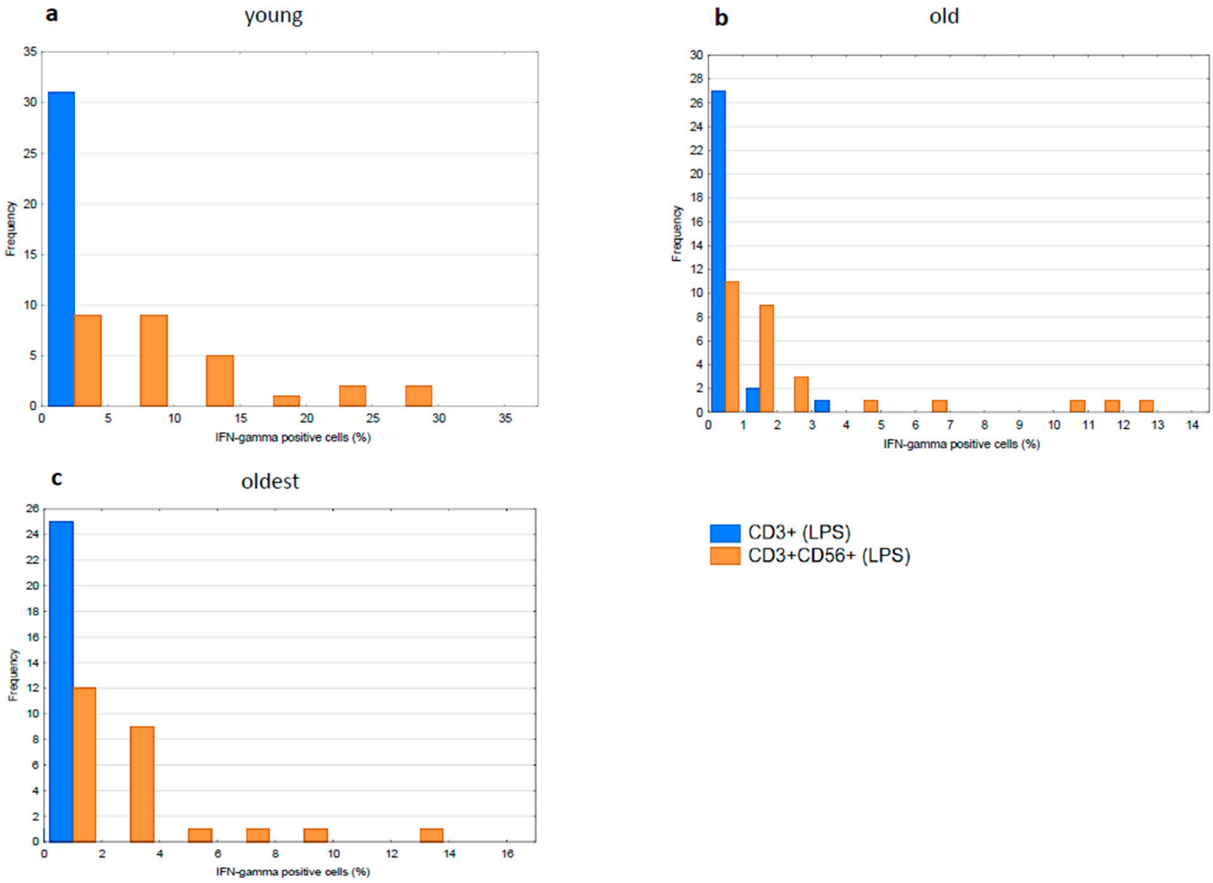

Figure S6. Frequency of samples differing in IFN-γ expression in the population of CD3+ cells (blue color) and CD3+CD56+ cells (orange color) stimulated with LPS analysed in the group of the young (**a**), old (**b**) and the oldest (**c**).

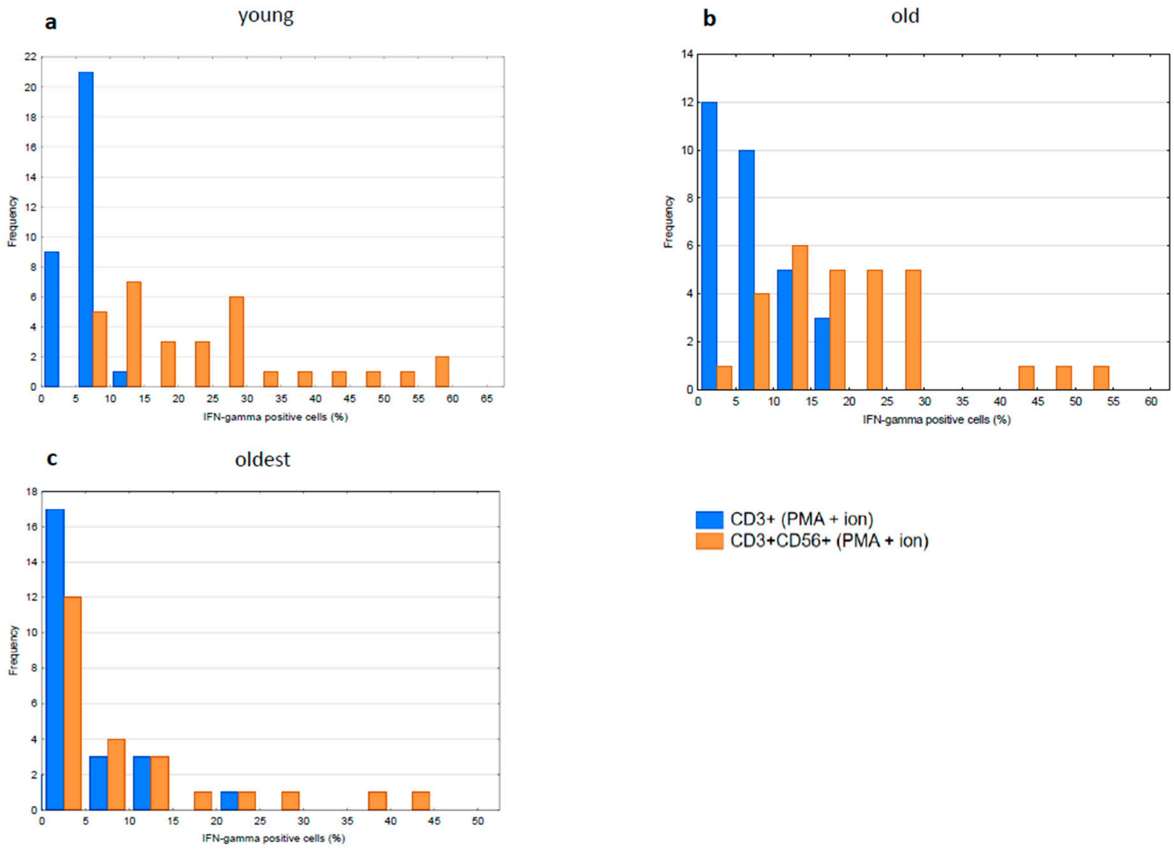

Figure S7. Frequency of samples differing in IFN-γ expression in the population of CD3+ cells (blue color) and CD3+CD56+ cells (orange color) stimulated with PMA and ionomycin analysed in the group of the young (**a**), old (**b**) and the oldest (**c**).
